# Understanding Attitudes and Perceptions Towards Blood Donation in Greece: An Analysis Aligned with the Health Belief Model

**DOI:** 10.1192/j.eurpsy.2024.1717

**Published:** 2024-08-27

**Authors:** M. Theodoratou, D. Papandrea, I. V. Papathanasiou, P. Andriopoulou, C. Dafogianni, I. Farmakopoulou

**Affiliations:** ^1^Social Sciences, Hellenic Open University, Patras, Greece; ^2^Health Sciences, Neapolis University Pafos, Pafos, Cyprus; ^3^Nursing, University of Thessaly, Larissa; ^4^Psychology, University of Ioannina, Ioannina; ^5^Nursing, University of West Attica, Athens; ^6^Social Work, University of Patras, Patras, Greece

## Abstract

**Introduction:**

This study explores attitudes toward blood donation in Greece, where maintaining an adequate supply is challenging. Using the Health Belief Model, we examine factors like perceived severity, vulnerability, and self-efficacy.

**Objectives:**

The main aims of this study are to assess public perceptions and barriers concerning blood shortage in Greece, and to identify motivators and self-efficacy levels for regular blood donation.

**Methods:**

A cross-sectional study was conducted using a self-administered questionnaire distributed to a sample of Greek adults. The questionnaire was designed based on the constructs of the Health Belief Model and included questions related to perceived severity, vulnerability, self-efficacy, and barriers and facilitators to blood donation. Descriptive statistics were used to analyse the responses, calculating means and standard deviations (SDs) for each variable.

**Results:**

Perceived Severity and Vulnerability

Participants in our study show a heightened awareness of the severity of blood shortages, especially in summer months and during increased surgical interventions. They also acknowledge Greece’s dependency on more than just voluntary donations to meet blood supply needs. These findings align with the Health Belief Model’s constructs of perceived severity and vulnerability, suggesting avenues for promoting donation.

Perceived Benefits and Barriers

A significant 74% of participants believe they can regularly donate blood and plan to do so in the next six months. However, fear of needles, health concerns, and fears of transmissible diseases act as barriers. According to the Health Belief Model, targeting these barriers could facilitate blood donation.

Self-Efficacy

A high percentage (74%, SD=5) of participants displayed strong self-efficacy, suggesting they are likely to engage in blood donation if encouraged. This aligns with the Health Belief Model’s emphasis on self-efficacy as a motivator for health actions.

Cues to Action

Participants identified informational campaigns, digital reminders, and social encouragement as cues to action, with a disfavor for financial incentives. These cues could serve as triggers for blood donation, consistent with the Health Belief Model.

**Conclusions:**

By aligning these findings with the Health Belief Model, it becomes evident that there are strong perceptions of severity and vulnerability, but also considerable barriers to overcome. The high self-efficacy among participants and the cues to action identified could serve as bases for targeted interventions to improve blood donation rates.

**Disclosure of Interest:**

None Declared

